# *N*-(2-Chloro-2,2-difluoro­acet­yl)-*N*′,*N*′′-diisopropyl­phospho­ric triamide

**DOI:** 10.1107/S1600536811005435

**Published:** 2011-02-19

**Authors:** Mehrdad Pourayoubi, Anahid Saneei

**Affiliations:** a Department of Chemistry, Ferdowsi University of Mashhad, Mashhad 91779, Iran

## Abstract

In the title compound, C_8_H_17_ClF_2_N_3_O_2_P, the phosphoryl group and the NH unit of the C(O)NHP(O) moiety adopt a *syn* conformation with respect to each other. The P atom is in a tetra­hedral coordination environment and the environment of the N atom of the C(O)NHP(O) moiety is essentially planar. In the crystal, adjacent mol­ecules are linked *via* N—H⋯O =P and N—H⋯O =C hydrogen bonds, building *R*_2_^2^(8) and *R*_2_^2^(12) rings in a linear arrangement parallel to [110].

## Related literature

For metal complexes of phosphoryl donor ligands, see: Gholivand *et al.* (2010 ▶). For a phospho­ric triamide compound having a C(=O)NHP(=O) skeleton, see: Pourayoubi *et al.* (2010 ▶). For hydrogen-bond motifs, see: Etter *et al.* (1990 ▶); Bernstein *et al.* (1995 ▶). For the synthesis of the starting material, CClF_2_C(O)NHP(O)Cl_2_, see: Iriarte *et al.* (2008 ▶).
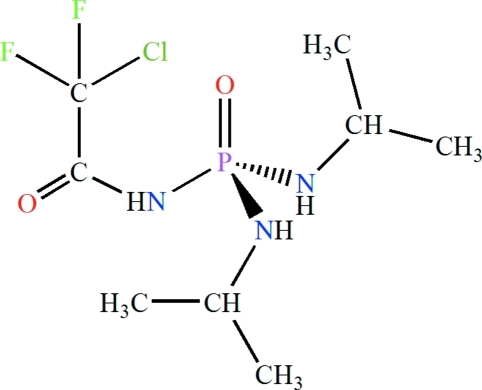


## Experimental

### 

#### Crystal data

C_8_H_17_ClF_2_N_3_O_2_P
*M* *_r_* = 291.67
Triclinic, 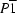

*a* = 8.1993 (7) Å
*b* = 9.6735 (9) Å
*c* = 9.8331 (9) Å
α = 99.784 (2)°
β = 105.999 (2)°
γ = 110.770 (2)°
*V* = 669.18 (10) Å^3^
*Z* = 2
Mo *K*α radiation
μ = 0.42 mm^−1^
*T* = 100 K
0.26 × 0.19 × 0.16 mm

#### Data collection

Bruker SMART APEXII CCD area-detector diffractometer
Absorption correction: multi-scan (*SADABS*; Bruker, 2005 ▶) *T*_min_ = 0.602, *T*_max_ = 0.750
9089 measured reflections
4218 independent reflections
3337 reflections with *I* > 2σ(*I*)
*R*_int_ = 0.024

#### Refinement

*R*[*F* ^2^ > 2σ(*F* ^2^)] = 0.036
*wR*(*F* ^2^) = 0.095
*S* = 1.04
4218 reflections
158 parameters
H-atom parameters constrained
Δρ_max_ = 0.56 e Å^−3^
Δρ_min_ = −0.30 e Å^−3^

### 

Data collection: *APEX2* (Bruker, 2005 ▶); cell refinement: *SAINT* (Bruker, 2005 ▶); data reduction: *SAINT*; program(s) used to solve structure: *SHELXS97* (Sheldrick, 2008 ▶); program(s) used to refine structure: *SHELXTL* (Sheldrick, 2008 ▶); molecular graphics: *Mercury* (Macrae *et al.*, 2008 ▶) and *PLATON* (Spek, 2009 ▶); software used to prepare material for publication: *SHELXL97* (Sheldrick, 2008 ▶).

## Supplementary Material

Crystal structure: contains datablocks global, I. DOI: 10.1107/S1600536811005435/dn2655sup1.cif

Structure factors: contains datablocks I. DOI: 10.1107/S1600536811005435/dn2655Isup2.hkl

Additional supplementary materials:  crystallographic information; 3D view; checkCIF report

## Figures and Tables

**Table 1:** Hydrogen-bond geometry (Å, °)

*D*—H⋯*A*	*D*—H	H⋯*A*	*D*⋯*A*	*D*—H⋯*A*
N1—H1⋯O2^i^	0.88	1.87	2.7295 (13)	164
N3—H3⋯O1^ii^	0.85	2.14	2.9645 (14)	163

Symmetry codes: (i) 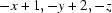; (ii) 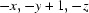.

**Fig. 1.**
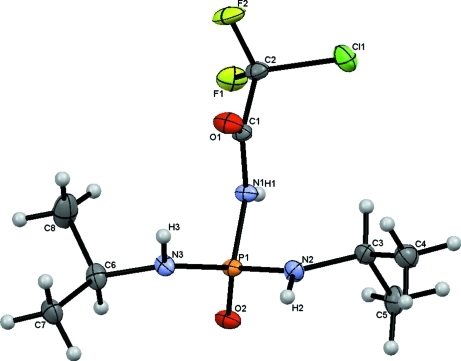
An ORTEP-style plot of title compound with the atom labeling scheme. Ellipsoids are shown at the 50% probability level. H atoms are represented as small spheres of arbitrary radii.

**Fig. 2.**
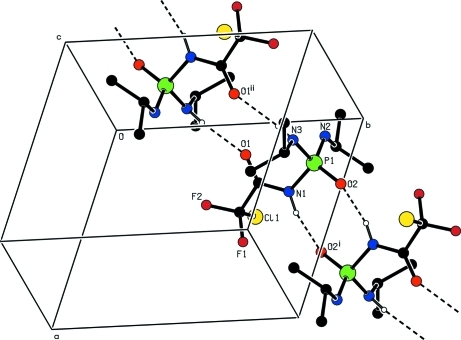
Partial packing view showing the formation of the chain through N-H···O hydrogen bonds which are shown as dashed lines. H atoms not involved in hydrogen bondings have been omitted for the sake of clarity. [Symmetry codes: (i) -x+1, -y+2, -z; (ii) -x, -y+1, -z]

## supplementary crystallographic information

### Comment

Carbacylamidophosphates with a –C(O)NHP(O)- skeleton have attracted attention because of their roles as the *O*,*O'*-donor ligands for metal complexation (Gholivand *et al.*, 2010). Following our previous works about phosphorus compounds containing C(O)NHP(O) moiety such as P(O)[NHC(O)C_6_H_3_(2,6-F_2_)][NHC(CH_3_)_3_]_2_ (Pourayoubi *et al.*, 2010), we report here on the synthesis and crystal structure of P(O)[NHC(O)CClF_2_][NH(C_3_H_7_)]_2_. Single crystals of title compound were obtained from a solution of CH_3_OH and CH_3_CN after a slow evaporation at room temperature.

The phosphoryl group and NH unit are *syn* to each other and the phosphorus atom has a slightly distorted tetrahedral configuration (Fig. 1). The bond angles around the P atom are in the range of 103.30 (6)° to 119.69 (6)°. The P—N2 and P—N3 bonds (with bond lengths of 1.6262 (12) Å and 1.6190 (11) Å) are shorter than the P—N1 bond (1.7039 (11) Å). The environment of nitrogen N1 atom is essentially planar. The P═O bond length of 1.4768 (9) Å is standard for phosphoramidate compounds.

In the crystal structure, adjacent molecules are linked *via* N—H···O ═P and N—H···O ═C hydrogen bonds, building *R*_2_^2^(8) and *R*_2_^2^(12) rings (Etter *et al.*, 1990; Bernstein *et al.*, 1995) in a linear arrangement parallel to the *ab* plane in the direction of [110] axis (Table 1, Fig. 2).

### Experimental

Synthesis of CClF_2_C(O)NHP(O)Cl_2_ CClF_2_C(O)NHP(O)Cl_2_ was prepared according to procedure reported by Iriarte *et al.* (2008) from a reaction between phosphorus pentachloride (16.91 mmol) and CClF_2_C(O)NH_2_ (16.91 mmol) in dry CCl_4_ at 358 K (3 h) and then the treatment of formic acid (16.91 mmol) at ice bath temperature; then removing of solvent in vacuum to yield CClF_2_C(O)NHP(O)Cl_2_.

Synthesis of title compound To a solution of CClF_2_C(O)NHP(O)Cl_2_ (2.09 mmol) in dry CHCl_3_, a solution of *N*-*iso*-propylamine (8.36 mmol) in dry CHCl_3_ was added dropwise and stirred at 273 K. After 4 h, the solvent was evaporated at room temperature. The solid was washed with H_2_O. The product was obtained after recrystallization from a methanol/acetonitrile mixture (4:1) after a slow evaporation at room temperature. IR (KBr, cm^-1^): 3400, 3057, 2910, 2890, 2730, 1740 (C═O), 1500, 1260, 1218, 1165, 1118, 1095, 978, 920, 840, 738, 720.

### Refinement

All H atoms attached to C atoms and the planar N1 atom were fixed geometrically and treated as riding with C—H = 0.98 Å (methyl) or 1.0 Å (methine) and N—H = 0.86 Å with U_iso_(H) = 1.2U_eq_(Cmethine or N) or U_iso_(H) = 1.5U_eq_(CH3). H atoms for N2 and N3 were located in difference Fourier maps and included in the subsequent refinement using restraints (N-H= 0.86 (1)Å with U_iso_(H) = 1.5U_eq_(N). In the last cycles of refinement they were treated as riding on their parent N atoms.

### Crystal data

**Table:**

C_8_H_17_ClF_2_N_3_O_2_P	*Z* = 2
*M**_r_* = 291.67	*F*(000) = 304
Triclinic, *P*1	*D*_x_ = 1.448 Mg m^−^^3^
Hall symbol: -P 1	Mo *K*α radiation, λ = 0.71073 Å
*a* = 8.1993 (7) Å	Cell parameters from 2867 reflections
*b* = 9.6735 (9) Å	θ = 2.3–30.9°
*c* = 9.8331 (9) Å	µ = 0.42 mm^−^^1^
α = 99.784 (2)°	*T* = 100 K
β = 105.999 (2)°	Prizm, colorless
γ = 110.770 (2)°	0.26 × 0.19 × 0.16 mm
*V* = 669.18 (10) Å^3^	

### Data collection

**Table:**

Bruker SMART APEXII CCD area-detector diffractometer	4218 independent reflections
Radiation source: fine-focus sealed tube	3337 reflections with *I* > 2σ(*I*)
graphite	*R*_int_ = 0.024
φ and ω scans	θ_max_ = 31.0°, θ_min_ = 2.3°
Absorption correction: multi-scan (*SADABS*; Bruker, 2005)	*h* = −11→11
*T*_min_ = 0.602, *T*_max_ = 0.750	*k* = −14→14
9089 measured reflections	*l* = −14→14

### Refinement

**Table:**

Refinement on *F*^2^	Primary atom site location: structure-invariant direct methods
Least-squares matrix: full	Secondary atom site location: difference Fourier map
*R*[*F*^2^ > 2σ(*F*^2^)] = 0.036	Hydrogen site location: inferred from neighbouring sites
*wR*(*F*^2^) = 0.095	H-atom parameters constrained
*S* = 1.04	*w* = 1/[σ^2^(*F*_o_^2^) + (0.0527*P*)^2^ + 0.0127*P*] where *P* = (*F*_o_^2^ + 2*F*_c_^2^)/3
4218 reflections	(Δ/σ)_max_ < 0.001
158 parameters	Δρ_max_ = 0.56 e Å^−^^3^
0 restraints	Δρ_min_ = −0.30 e Å^−^^3^

### Special details

**Table:**

Geometry. All esds (except the esd in the dihedral angle between two l.s. planes) are estimated using the full covariance matrix. The cell esds are taken into account individually in the estimation of esds in distances, angles and torsion angles; correlations between esds in cell parameters are only used when they are defined by crystal symmetry. An approximate (isotropic) treatment of cell esds is used for estimating esds involving l.s. planes.
Refinement. Refinement of *F*^2^ against ALL reflections. The weighted *R*-factor *wR* and goodness of fit *S* are based on *F*^2^, conventional *R*-factors *R* are based on *F*, with *F* set to zero for negative *F*^2^. The threshold expression of *F*^2^ > σ(*F*^2^) is used only for calculating *R*-factors(gt) *etc*. and is not relevant to the choice of reflections for refinement. *R*-factors based on *F*^2^ are statistically about twice as large as those based on *F*, and *R*- factors based on ALL data will be even larger.

### Fractional atomic coordinates and isotropic or equivalent isotropic displacement parameters (Å^2^)

**Table:**

	*x*	*y*	*z*	*U*_iso_*/*U*_eq_	
P1	0.23257 (4)	0.86972 (4)	0.03714 (4)	0.01390 (8)	
Cl1	0.30477 (6)	0.56630 (4)	−0.36890 (4)	0.03081 (10)	
F1	0.54717 (11)	0.63181 (10)	−0.11562 (10)	0.0284 (2)	
F2	0.31465 (14)	0.40675 (9)	−0.19141 (11)	0.0322 (2)	
O1	0.10801 (14)	0.52987 (10)	−0.10574 (11)	0.0238 (2)	
O2	0.37834 (13)	1.03015 (10)	0.10823 (10)	0.0196 (2)	
N1	0.33089 (14)	0.77459 (12)	−0.05183 (12)	0.0146 (2)	
H1	0.4368	0.8292	−0.0609	0.018*	
N2	0.03816 (15)	0.83557 (13)	−0.09290 (12)	0.0182 (2)	
H2	−0.0483	0.8420	−0.0661	0.022*	
N3	0.16459 (15)	0.79008 (12)	0.15566 (12)	0.0155 (2)	
H3	0.0753	0.6993	0.1225	0.019*	
C1	0.25344 (17)	0.61960 (14)	−0.10878 (14)	0.0153 (2)	
C2	0.36184 (19)	0.55503 (15)	−0.18656 (15)	0.0192 (3)	
C3	0.02381 (19)	0.84469 (16)	−0.24394 (15)	0.0201 (3)	
H3A	0.0704	0.7721	−0.2874	0.024*	
C4	−0.1816 (2)	0.78961 (18)	−0.33779 (16)	0.0259 (3)	
H4A	−0.2525	0.6837	−0.3386	0.039*	
H4B	−0.1939	0.7916	−0.4394	0.039*	
H4C	−0.2309	0.8581	−0.2959	0.039*	
C5	0.1427 (2)	1.00657 (18)	−0.24315 (16)	0.0262 (3)	
H5A	0.2750	1.0341	−0.1889	0.039*	
H5B	0.1051	1.0809	−0.1947	0.039*	
H5C	0.1246	1.0090	−0.3453	0.039*	
C6	0.29579 (18)	0.82087 (16)	0.30596 (15)	0.0208 (3)	
H6A	0.3976	0.9277	0.3361	0.025*	
C7	0.1941 (2)	0.81568 (18)	0.41385 (16)	0.0268 (3)	
H7A	0.1366	0.8888	0.4081	0.040*	
H7B	0.2834	0.8442	0.5148	0.040*	
H7C	0.0965	0.7108	0.3884	0.040*	
C8	0.3848 (3)	0.7080 (2)	0.3090 (2)	0.0390 (4)	
H8A	0.4568	0.7195	0.2437	0.058*	
H8B	0.2867	0.6020	0.2749	0.058*	
H8C	0.4685	0.7294	0.4105	0.058*	

### Atomic displacement parameters (Å^2^)

**Table:**

	*U*^11^	*U*^22^	*U*^33^	*U*^12^	*U*^13^	*U*^23^
P1	0.01625 (15)	0.00992 (14)	0.01683 (16)	0.00349 (11)	0.01039 (12)	0.00433 (11)
Cl1	0.0425 (2)	0.0345 (2)	0.02119 (18)	0.01780 (18)	0.01803 (16)	0.00824 (15)
F1	0.0208 (4)	0.0305 (5)	0.0358 (5)	0.0133 (4)	0.0117 (4)	0.0065 (4)
F2	0.0469 (6)	0.0162 (4)	0.0489 (6)	0.0186 (4)	0.0300 (5)	0.0147 (4)
O1	0.0247 (5)	0.0128 (4)	0.0318 (5)	0.0005 (4)	0.0187 (4)	0.0040 (4)
O2	0.0237 (5)	0.0115 (4)	0.0233 (5)	0.0026 (4)	0.0158 (4)	0.0030 (4)
N1	0.0148 (5)	0.0108 (5)	0.0195 (5)	0.0034 (4)	0.0109 (4)	0.0043 (4)
N2	0.0203 (5)	0.0223 (5)	0.0191 (5)	0.0112 (5)	0.0126 (4)	0.0091 (4)
N3	0.0153 (5)	0.0125 (5)	0.0161 (5)	0.0012 (4)	0.0081 (4)	0.0042 (4)
C1	0.0178 (6)	0.0119 (5)	0.0171 (6)	0.0054 (5)	0.0089 (5)	0.0047 (4)
C2	0.0243 (7)	0.0134 (6)	0.0246 (7)	0.0089 (5)	0.0131 (5)	0.0076 (5)
C3	0.0255 (7)	0.0244 (7)	0.0177 (6)	0.0151 (6)	0.0118 (5)	0.0076 (5)
C4	0.0262 (7)	0.0281 (7)	0.0222 (7)	0.0131 (6)	0.0066 (6)	0.0045 (6)
C5	0.0276 (7)	0.0329 (8)	0.0226 (7)	0.0121 (6)	0.0129 (6)	0.0148 (6)
C6	0.0176 (6)	0.0220 (6)	0.0180 (6)	0.0032 (5)	0.0057 (5)	0.0069 (5)
C7	0.0352 (8)	0.0334 (8)	0.0180 (6)	0.0181 (7)	0.0124 (6)	0.0099 (6)
C8	0.0395 (9)	0.0669 (12)	0.0355 (9)	0.0384 (9)	0.0212 (8)	0.0278 (9)

### Geometric parameters (Å, °)

**Table:**

P1—O2	1.4768 (9)	C3—H3A	1.0000
P1—N3	1.6190 (11)	C4—H4A	0.9800
P1—N2	1.6262 (12)	C4—H4B	0.9800
P1—N1	1.7039 (11)	C4—H4C	0.9800
Cl1—C2	1.7566 (14)	C5—H5A	0.9800
F1—C2	1.3366 (16)	C5—H5B	0.9800
F2—C2	1.3351 (15)	C5—H5C	0.9800
O1—C1	1.2127 (15)	C6—C8	1.513 (2)
N1—C1	1.3447 (15)	C6—C7	1.5164 (19)
N1—H1	0.8800	C6—H6A	1.0000
N2—C3	1.4775 (17)	C7—H7A	0.9800
N2—H2	0.8390	C7—H7B	0.9800
N3—C6	1.4754 (17)	C7—H7C	0.9800
N3—H3	0.8536	C8—H8A	0.9800
C1—C2	1.5399 (17)	C8—H8B	0.9800
C3—C5	1.520 (2)	C8—H8C	0.9800
C3—C4	1.5207 (19)		
			
O2—P1—N3	112.27 (5)	C3—C4—H4A	109.5
O2—P1—N2	119.69 (6)	C3—C4—H4B	109.5
N3—P1—N2	103.94 (6)	H4A—C4—H4B	109.5
O2—P1—N1	105.13 (5)	C3—C4—H4C	109.5
N3—P1—N1	112.31 (5)	H4A—C4—H4C	109.5
N2—P1—N1	103.30 (6)	H4B—C4—H4C	109.5
C1—N1—P1	122.76 (9)	C3—C5—H5A	109.5
C1—N1—H1	118.6	C3—C5—H5B	109.5
P1—N1—H1	118.6	H5A—C5—H5B	109.5
C3—N2—P1	123.77 (9)	C3—C5—H5C	109.5
C3—N2—H2	116.2	H5A—C5—H5C	109.5
P1—N2—H2	117.0	H5B—C5—H5C	109.5
C6—N3—P1	121.89 (8)	N3—C6—C8	111.29 (12)
C6—N3—H3	112.7	N3—C6—C7	109.51 (11)
P1—N3—H3	117.9	C8—C6—C7	111.49 (12)
O1—C1—N1	126.01 (12)	N3—C6—H6A	108.1
O1—C1—C2	118.79 (11)	C8—C6—H6A	108.1
N1—C1—C2	115.19 (10)	C7—C6—H6A	108.1
F2—C2—F1	107.66 (11)	C6—C7—H7A	109.5
F2—C2—C1	109.72 (10)	C6—C7—H7B	109.5
F1—C2—C1	112.14 (11)	H7A—C7—H7B	109.5
F2—C2—Cl1	108.56 (10)	C6—C7—H7C	109.5
F1—C2—Cl1	108.94 (9)	H7A—C7—H7C	109.5
C1—C2—Cl1	109.74 (9)	H7B—C7—H7C	109.5
N2—C3—C5	111.99 (11)	C6—C8—H8A	109.5
N2—C3—C4	108.48 (11)	C6—C8—H8B	109.5
C5—C3—C4	112.00 (12)	H8A—C8—H8B	109.5
N2—C3—H3A	108.1	C6—C8—H8C	109.5
C5—C3—H3A	108.1	H8A—C8—H8C	109.5
C4—C3—H3A	108.1	H8B—C8—H8C	109.5
			
O2—P1—N1—C1	168.43 (10)	O1—C1—C2—F2	−22.92 (17)
N3—P1—N1—C1	46.05 (12)	N1—C1—C2—F2	158.19 (11)
N2—P1—N1—C1	−65.33 (11)	O1—C1—C2—F1	−142.50 (13)
O2—P1—N2—C3	72.29 (12)	N1—C1—C2—F1	38.60 (15)
N3—P1—N2—C3	−161.47 (10)	O1—C1—C2—Cl1	96.28 (13)
N1—P1—N2—C3	−44.04 (11)	N1—C1—C2—Cl1	−82.62 (12)
O2—P1—N3—C6	−36.29 (12)	P1—N2—C3—C5	−61.53 (14)
N2—P1—N3—C6	−167.07 (10)	P1—N2—C3—C4	174.35 (9)
N1—P1—N3—C6	81.94 (11)	P1—N3—C6—C8	−89.23 (13)
P1—N1—C1—O1	0.56 (19)	P1—N3—C6—C7	147.05 (10)
P1—N1—C1—C2	179.37 (9)		

### Hydrogen-bond geometry (Å, °)

**Table:**

*D*—H···*A*	*D*—H	H···*A*	*D*···*A*	*D*—H···*A*
N1—H1···O2^i^	0.88	1.87	2.7295 (13)	164
N3—H3···O1^ii^	0.85	2.14	2.9645 (14)	163

Symmetry codes: (i) −*x*+1, −*y*+2, −*z*; (ii) −*x*, −*y*+1, −*z*.
